# ﻿A new species of *Nototriche* (Malvaceae, Malvoideae) from the high Andes of Ecuador

**DOI:** 10.3897/phytokeys.261.157043

**Published:** 2025-08-05

**Authors:** Emilio J. Trujillo, David A. Espinel-Ortiz, Katya Romoleroux, Priscilla Muriel

**Affiliations:** 1 Herbario QCA, Facultad de Ciencias Exactas y Naturales, Pontificia Universidad Católica del Ecuador, Av. 12 de Octubre 1076 y Vicente Ramón Roca, 170525 Quito, Ecuador Pontificia Universidad Católica del Ecuador Quito Ecuador; 2 Bonn Institute of Organismic Biodiversity, University of Bonn, Meckenheimer Allee 170, 53115 Bonn, Germany University of Bonn Bonn Germany

**Keywords:** Antisana, Ecuador, Malvaceae, *Nototriche*, páramo, taxonomy

## Abstract

A new species of *Nototriche* (Malvaceae, Malvoideae) growing in the high Andes of Ecuador is described. *Nototricheantisanensis* E.J.Trujillo, Muriel, Espinel-Ortiz & Romol. is a cushion plant known only from the Antisana volcano in northeastern Ecuador. Morphologically, it is most similar to *N.jamesonii* A.W.Hill, but it differs in its cushion habit and vegetative characters, such as indumentum, corolla tube size, stamen head morphology, and the number of mericarps per fruit. Photographs of living plants and a scientific illustration of the new species are provided. In addition, a distribution map, an identification key, and a table comparing the habit, morphology, and distribution of all *Nototriche* species known from Ecuador are included.

## ﻿Introduction

*Nototriche* Turcz. (Malvaceae, Malvoideae) is a genus endemic to the Andes, with 100–120 species found from Ecuador to Argentina (Mazzei et al. 2024; Ulloa-Ulloa et al. 2025), and possibly Colombia. Within the Malvoideae, Colli-Silva et al. (2025) placed the genus in tribe Malveae and subtribe Malvinae, despite the lack of an epicalyx—shared by almost all other members of the subtribe—which Tate et al. (2005) argued was a secondary loss and an independent event. The genus most closely related to *Nototriche* is *Tarasa* Phil., and although both genera are currently recognized, their boundaries are still not completely resolved and require further study (Tate et al. 2005). All *Nototriche* species are herbs or prostrate shrubs that form lax or dense rosettes of basal leaves, which can be shortly caulescent. The stems are woody, sometimes with several branches, and can grow in soil, wind-blown dust, or sand. The leaves vary in shape, but in Ecuador, they share a pinnati – or palmati-lobed form with five or more lobes. The stipules are adnate to the petiole, forming a protective sheath called a vagina (Hill 1909). Flowers are solitary, and sometimes the pedicel arises at the center of the petiole. Fruits are schizocarps and are densely covered by various types of indumentum (Hill 1906; Krapovickas 1951; Fryxell 1992, 1997).

In Ecuador, *Nototriche* is found only in the páramo region of high-altitude mountains. Páramos are alpine ecosystems of the northern Andes and among the most biodiverse regions in the world, with high levels of endemism and speciation (Romoleroux et al. 2023). In general, alpine ecosystems occur on all continents above the tree-line ecotone and are characterized by extreme climatic conditions and specialized biodiversity adapted to them (Körner 2003). It is therefore not surprising that all *Nototriche* species found in Ecuador are endemic and adapted to local environmental conditions.

Fryxell (1992) was the first to revise the Ecuadorian species of *Nototriche*, and he recognized three species: *N.ecuadoriensis* Fryxell, *N.jamesonii* A.W.Hill, and *N.phyllanthos* (Cav.) A.W.Hill. He treated *N.hartwegii* A.W.Hill as a synonym of *N.jamesonii*. Jørgensen and León-Yanez (1999) recognized the same three species as occurring in Ecuador but did not mention *N.hartwegii*. When Chanco and Ulloa-Ulloa (2004) revisited the Ecuadorian species of the genus, they reestablished *N.hartwegii* as a species distinct from *N.jamesonii*, provided new descriptions, established the conservation status, and updated the distribution for each of the four species they recognized. Since then, the genus in Ecuador has received little attention, and given the limited amount of material examined by Fryxell (1992) and Chanco and Ulloa-Ulloa (2004), an updated revision using more specimens and additional techniques is warranted.

As part of a systematic revision of the Ecuadorian species, we identified a group of specimens misidentified as *Nototrichephyllanthos* or *N.jamesonii* that did not match any known species of *Nototriche* from Ecuador or neighboring Peru (Trujillo et al., in prep.). After an exhaustive morphological and literature review, we concluded that these specimens represent a new species, which is described below.

## ﻿Materials and methods

In January 2025, we collected material of *Nototricheantisanensis* from the Antisana volcano, near Antisanilla Peak at Laguna de Santa Lucía (00°28.436'S, 78°11.407'W). We carefully examined the collected specimens and analyzed additional gatherings of the genus housed at HA, QCA, and QCNE (Suppl. material 1). In addition, we examined online images of type material available from several herbaria (BM, BR, G, K, LD, NY, P, S, and US) and the JSTOR Global Plants database (https://plants.jstor.org/). We measured vegetative and reproductive characters of 92 collections, primarily from dried material, except for floral traits, for which measurements were taken from flowers rehydrated using Pohl’s solution (i.e., a solution of 750 ml of distilled water, 250 ml of 1-propanol, and 2 ml of liquid detergent). Traits were selected based on Hill’s (1909) monograph and Mazzei’s (2025) treatment of Peruvian taxa, both of which provide detailed reviews of the diagnostic characteristics of the genus. We further refined their character lists by identifying the less variable traits. Botanical terms used in the descriptions follow those of Stearn (1986) (proportions of length:width), and pubescence types are based on the terminology provided by Hickey and King (2000). Additionally, we consulted original publications and online images of the genus from the following bibliographical and nomenclatural sources: Biodiversity Heritage Library (https://www.biodiversitylibrary.org/), International Plant Names Index (IPNI 2025), Plants of the World Online (POWO 2025), and Tropicos (2025). We described the species and designated types following the "International Code of Nomenclature for algae, fungi, and plants" (Turland et al. 2025).

## ﻿Taxonomic treatment

### ﻿ Nototriche antisanensis

Taxon classificationPlantaeMalvalesMalvaceae

E.J.Trujillo, Muriel, Espinel-Ortiz & Romol. sp. nov.

9C4B0E2C-17E2-5E59-BE90-7825DB5A593E

urn:lsid:ipni.org:names:77366491-1

Figs 1 , 2 , 3 , 4

#### Diagnosis.

*Nototricheantisanensis* is morphologically most similar to *N.jamesonii* in having both surfaces of the leaf lamina covered by small stellate trichomes, triangular stipules and connate petals but the former differs from the latter in its cushion-forming (*vs.* prostrate shrubby) habit, dense (*vs.* lax) rosettes with 3 (*vs.* 9) lobes, free part of the stipule narrowly triangular with a 6:1 proportion (*vs.* triangular with a 2:1 proportion), sheath with both faces covered by stellate indumentum (*vs.* abaxial face glabrous), flowers with corolla tubes 1–2 mm (*vs.* 2.5–5 mm) long, anthers forming a globose head with a spherical 1:1 proportion, 2–3 × 2–3 mm (*vs.* elliptic 2:1 proportion, 3–5.5 × 2.2–3 mm); and a fruit with 11 (*vs.* 10) mericarps.

**Figure 1.**
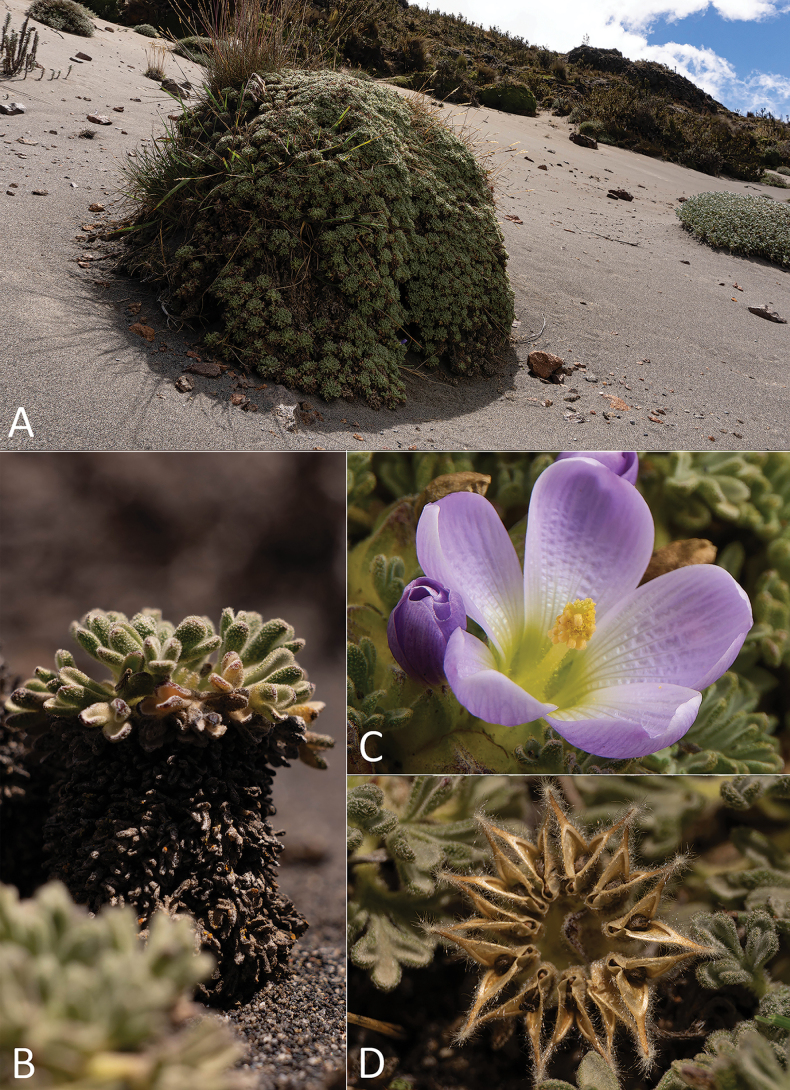
*Nototricheantisanensis*, from the collection *E.J Trujillo et al. 41*. **A.** Habit; **B.** Leaves in rosette; **C.** Top view of a flower; **D.** Fruit. Photographs by Erick Troncoso-López.

#### Type.

Ecuador • Napo: Cara oeste del volcán Antisana, alrededores de la Laguna de Santa Lucía, arenal en la cara norte del pico Antisanilla, 00°28.436'S, 78°11.407'W, 4325 m elev., 18 Jan 2025 (fl), *E.J. Trujillo R. Barrera-Cabezas, G. Núñez & N. Carvajal 41* (Holotype: QCA [barcode-252205]; isotype: QCA [barcode-254675]).

**Figure 2.**
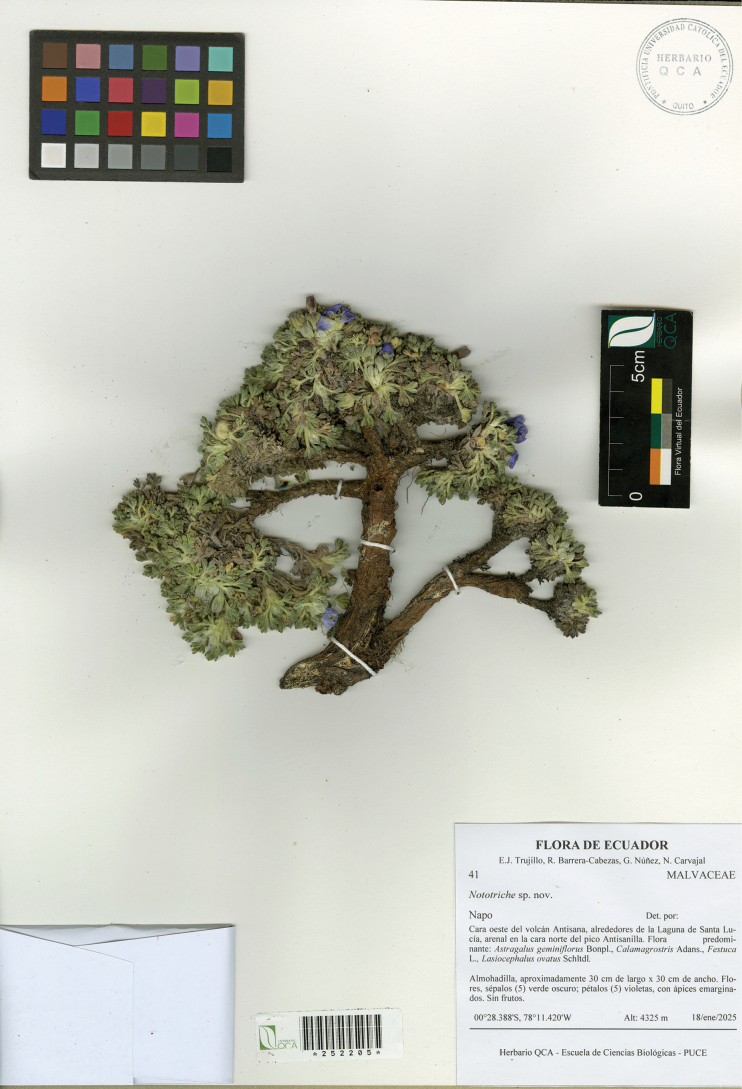
*Nototricheantisanensis* E.J. Trujillo, Muriel, Espinel-Ortiz & Romol. Holotype specimen (*E.J. Trujillo et al. 41*, QCA [barcode-252205]).

#### Description.

***Shrubs*** with short ***caulescent*** leaves forming cushions with subterranean woody stems with several ramifications. ***Leaves*** palmati-lobed; leaf blade 5.7–7.7 × 6.5–12.8 mm, trilobate with three subdivisions in each lobe; adaxial surface densely covered by stellate trichomes with rays 0.1–0.2 mm long; abaxial surface sparsely covered by stellate trichomes with rays 0.1–0.3 mm long. ***Stipules*** adnate to the petioles, forming a protective sheath, 4–7.1 × 2–3 mm, with stellate trichomes on both sides and along the margin, rays 0.3–0.7 mm long; free part of the stipule narrowly triangular, 2–4.5 × 1 mm, with stellate trichomes, rays 0.2–0.6 mm long, on both sides of the stipule. ***Free part of the petioles*** 4.5–7 × 0.6–1.2 mm, pubescent on both sides, with stellate trichomes, rays 0.1–0.4 mm long. ***Flowers*** 14–18 × 9–12 mm. Calyx 7.5–9.6 × 5.4–8.8 mm; sepals 5, connate, free at the apex forming triangular teeth, 3.1–4.8 × 2.1–3.1 mm, upper surface densely covered by stellate trichomes with rays 0.2–0.5 mm long, lower surface covered only at the apex, forming a triangle of interspersed stellate and bifid trichomes, rays 0.6–1.1 mm long; nectaries 5, transversely elliptic, distributed at the basal part of each sepal, 0.7–1 × 0.5–1 mm. Corolla slightly campanulate, violet with some degradation to white at the basal part of the petals, corolla tube 1–2 × 2–2.5 mm; petals 5, obovate, slightly emarginate, free part of the petals 11–13.6 × 5.5–7.28 mm, densely covered at the base by stellate, villous, and bifid trichomes, rays 0.6–1 mm long. Staminal tube 6–8 × 0.5–0.7 mm, covered with stellate and bifid trichomes, rays 0.5–1.5 mm long; anthers forming a spherical globose head 2–3 × 2–3 mm, covering 1/3 of the staminal tube length. Carpels with 11 styles, stigmas capitate. ***Fruit*** a schizocarp with 11 mericarps; each mericarp 5.5 × 2.5 mm, densely covered by stellate trichomes with rays ca. 1 mm long. ***Seeds*** kidney-shaped, 2 × 2 mm, one seed per mericarp.

**Figure 3.**
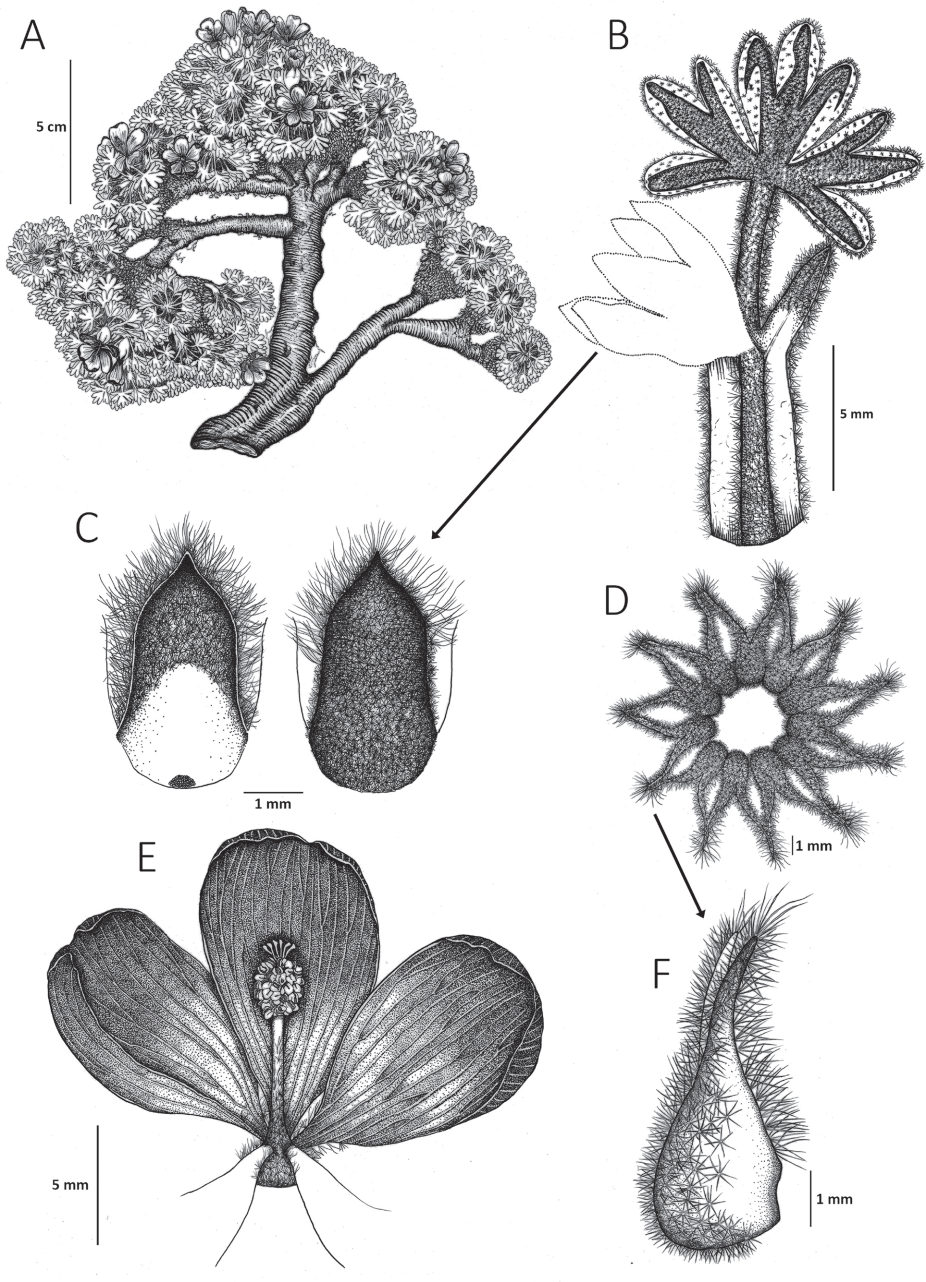
*Nototricheantisanensis* E.J. Trujillo, Muriel, Espinel-Ortiz & Romol. **A.** Habit; **B.** Leaf; **C.** Lower surface (left) and upper surface (right) of the sepals; **D.** Fruit; **E.** Corolla; **F.** Isolated mericarp. (**A–C, E.** Based on *E.J. Trujillo et al. 41* (QCA); **D, F.** Based on *E.J. Trujillo et al. 50* (QCA)). Illustrations by Carla J. Rodríguez.

#### Additional specimens examined.

**Ecuador** • **Napo**: Antisana, western side of the volcano, cushion bog [sic] inside the Antisanilla crater, 00°28'18"S, 78°11'32"W, 4300 m elev., 22 Nov 2018, *P. Sklenář et al. 15876* (QCA); • Alrededores de la Laguna de Santa Lucía, arenal en la cara norte del pico Antisanilla, 00°28.436'S, 78°11.407'W, 4325 m elev., 18 Jan 2025 (fl), *E.J. Trujillo et al. 40* (QCA); • Ibid., 00°28.447'S, 78°11.404'W, 4380 m elev., 18 Jan 2025 (fl), *E.J. Trujillo et al. 43* (QCA); • Ibid., 00°28.400'S, 78°11.425'W, 4352 m elev., 18 Jan 2025 (fl), *E.J. Trujillo et al. 44* (QCA); • Ibid., *E.J. Trujillo et al. 45* (QCA); • Ibid., 00°28.388'S, 78°11.420'W, 4352 m elev., 18 Jan 2025, *E.J. Trujillo et al. 46* (QCA); • Ibid., *E.J. Trujillo et al. 47* (QCA); • Ibid., *E.J. Trujillo et al. 48* (QCA); • Ibid., *E.J. Trujillo et al. 49* (QCA); • Ibid., *E.J. Trujillo et al. 50* (QCA).

#### Distribution, habitat, and ecology.

**Figure 4.**
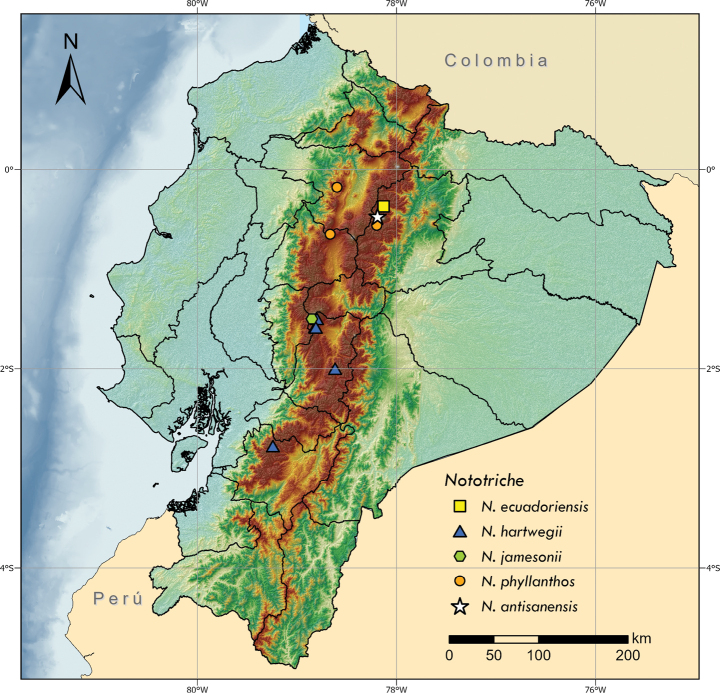
Distribution of *Nototriche* species in the Andes of Ecuador.

*Nototricheantisanensis* is only known from the type locality on the Antisana volcano in the northeastern Andes of Ecuador (Fig. 4). It grows in arid and sandy soils behind a peak that protects individual plants from strong winds and freezing. Characteristic taxa growing nearby include *Astragalusgeminiflorus* Bonpl. (Fabaceae), *Lasiocephalusovatus* Schltdl. (Asteraceae), and species of *Calamagrostis* Adans. and *Festuca* L. (Poaceae).

#### Phenology.

Recorded flowering in October, November, and January. Fruiting in November.

#### Etymology.

The specific epithet honors the páramos of the Antisana volcano, where this species was found and collected for the first time.

#### Preliminary assessment of conservation status.

*Nototricheantisanensis* is only known from several individuals (<50 mature individuals) growing around the type locality at Antisanilla Peak, in the páramos of the Antisana National Park. Its distribution appears to be restricted to this area due to specific ecological characteristics. Extensive sampling across other páramos yielded no additional records of this species in similarly arid environments. Furthermore, several threats have been identified, including the expansion of the agricultural frontier, climate change, and the construction of infrastructure related to renewable energy projects in nearby cities. Based on the available information and following the IUCN Red List Criteria and Guidelines (IUCN 2024), *N.antisanensis* is preliminarily assessed as Critically Endangered (CR).

#### Discussion.

The taxonomy of *Nototriche* is challenging due to the lack of comprehensive studies and the phenotypic plasticity exhibited by its species in response to extreme environmental conditions. Despite these difficulties, Hill (1906, 1909, 1928, 1932, 1933) and Krapovickas (1950, 1951, 1953, 1957, 1973) provided most of the species descriptions and established key diagnostic characteristics that facilitate differentiation. Among these are the presence and size of the corolla tube, the distribution and morphology of the indumentum, and leaf morphology. Fruit characters and stamen morphology are particularly relevant for distinguishing taxa.

*Nototricheantisanensis* shares common characteristics with other species of the genus, such as palmati-lobed leaves with multiple subdivisions arranged in a rosette, axillary flowers, stipules adnate to the petiole, nectaries at the base of the sepals, and a dense presence of trichomes on the corolla. Due to certain morphological traits (see diagnosis above) and some ecological features (such as growing in sandy soils), this species was initially misidentified as *N.jamesonii* in a herbarium voucher at QCA. However, it is clear that *N.jamesonii* is restricted to the western arid páramos of the Chimborazo volcano and exhibits several morphological differences, as outlined in the diagnosis of *N.antisanensis*. Additionally, this new species is distinct from other *Nototriche* species based on several key characteristics (Table 1), such as the corolla tube and vagina indumentum.

**Table 1.** Table comparing morphological differences among the five species of *Nototriche* known from Ecuador.

	*N.antisanensis*	*N.ecuadoriensis*	*N.jamesonii*	*N.phyllanthos*	*N.hartwegii*
**Habit**	Cushion	Acaulescent herb	Prostrate shrub	Prostrate shrub	Cushion
**Leaf lamina morphology**	Palmati-lobed, tri-lobate, with 3 obtuse subdivisions in each lobe	Pinnati-lobed, trilobate, with 5–10 acute subdivisions in each lobe	Palmati-lobed, trilobate, with ≥ 3 obtuse subdivisions in each lobe	Palmati-lobed, trilobate with ≥ 3 obtuse subdivisions in each lobe	Palmati-lobed, trilobate with obtuse lobes
**Leaf lamina indumentum**	Stellate, rays 0.1–0.3 mm long, present on both surfaces of the leaf blade	Villous, rays 0.5–1.4 mm long, present only at the tips of the lobes	Stellate, rays 0.1–0.4 mm long, present on both surfaces of the leaf blade	Stellate, rays 0.1–0.8 mm long, present on the adaxial face of the leaf blade, abaxial face glabrous	Stellate, rays 0.8–1.2 mm long, present on both surfaces of the leaf blade
**Free part of the stipules**	Narrowly triangular	Slightly triangular with an obtuse apex	Triangular	Slightly triangular	Oblanceolate
**Sheath indumentum**	Stellate, on both sides of the vagina	Glabrous	Stellate, only on the adaxial side of the vagina	Stellate, on both sides of the vagina	Stellate, on both sides of the vagina
**Corolla tube**	Present, 1–2 mm long	Present, 4 mm long	Present, 2.5–4 mm long	Absent	Absent
**Stamen head**	Globose (1:1 proportion)	Spherical globose (1:1 proportion)	Elliptic (2:1 proportion)	Elliptic (2:1 proportion)	Elliptic (2:1 proportion)
**Fruit**	11 mericarps, 5.5 × 2.5 mm	No Data	10 mericarps, 6 × 2.5 mm	7 mericarps, 7 × 1.5 mm	12 mericarps, 9 × 3 mm
**Distribution**	Restricted to the Antisana páramos in the northeastern Andes of Ecuador	In the northeastern Andes of Ecuador	Restricted to the central Andes of Ecuador	Ranging from the central to the northern Andes of Ecuador	From the southern to the central Andes of Ecuador

The corolla tube is a crucial diagnostic feature of the genus (Hill 1909) and serves as the first-step characteristic for distinguishing these five species. *Nototrichephyllanthos* and *N.hartwegii* are the only two species in which this structure is absent (they possess free petals), differing from each other by indumentum length and presence. *Nototrichephyllanthos* has a glabrous abaxial leaf surface, whereas *N.hartwegii* has abundant stellate trichomes on both surfaces of the leaf blade. Additionally, *N.phyllanthos* possesses slightly triangular stipules with a glabrous abaxial surface, while *N.hartwegii* has oblanceolate stipules that are densely pubescent on both sides.

The other three species, *Nototricheantisanensis*, *N.jamesonii*, and *N.ecuadoriensis*, have connate petals forming a corolla tube and require additional characteristics for proper identification. *Nototricheecuadoriensis* is the easiest to distinguish due to its glabrous leaf blades, except for the tips of the lobes, which are villous. In contrast, as specified in the diagnosis, *N.antisanensis* and *N.jamesonii* share similar indumentum distribution, type, and size but differ in growth habit (cushion vs. prostrate shrub), rosette morphology (compact vs. lax), corolla tube length (1–2 mm vs. 2.5–4 mm), and fruit composition (11 mericarps vs. 10 mericarps).

### ﻿Key to the Ecuadorian species of *Nototriche*

**Table:** 

1	Corolla tube present	**2**
–	Corolla tube absent	**4**
2	Leaf lamina pinnati-lobed, with presence of villous trichomes	***N.ecuadoriensis***
–	Leaf lamina palmati-lobed, with presence of stellate trichomes	**3**
3	Corolla tube 1–2 mm long, staminal tube with a spherical globose head	***N.antisanensis***
–	Corolla tube 2.5–4 mm long, staminal tube with an elliptical head	***N.jamesonii***
4	Adaxial surface of the leaf blade densely covered by short stellate trichomes, abaxial leaf blades glabrous, lobes with an acute apex, stipules triangular, mericarps 9, prostrate shrubby habit	***N.phyllanthos***
–	Adaxial and abaxial surfaces of the leaf lamina densely covered by long stellate trichomes, lobes with an obtuse apex, stipules oblanceolate, mericarps 12, cushion habit	***N.hartwegii***

## Supplementary Material

XML Treatment for Nototriche antisanensis

## ﻿Additional information

### Use of AI

No use of AI was reported.

### Author ORCIDs

Emilio J. Trujillo https://orcid.org/0009-0001-7725-3626

David A. Espinel-Ortiz https://orcid.org/0000-0003-3405-0375

Katya Romoleroux https://orcid.org/0000-0002-0679-9218

Priscilla Muriel https://orcid.org/0000-0002-7874-8057

### Data availability

All of the data that support the findings of this study are available in the main text or Supplementary Information.

## Supplementary materials

Supplementary material 1 Specimens examined of Ecuadorian *Nototriche* apart from *N.antisanensis*, the latter listed in the text Data type docx https://binary.pensoft.net/file/1388285This dataset is made available under the Open Database License (http://opendatacommons.org/licenses/odbl/1.0/). The Open Database License (ODbL) is a license agreement intended to allow users to freely share, modify, and use this Dataset while maintaining this same freedom for others, provided that the original source and author(s) are credited.Emilio J. Trujillo, David A. Espinel-Ortiz, Katya Romoleroux, Priscilla Muriel
