# Supplementary material for: ﻿A new species of Nototriche (Malvaceae, Malvoideae) from the high Andes of Ecuador
Source: PhytoKeys. 2025 Aug 5;261:1–12. doi: 10.3897/phytokeys.261.157043 (PMC12344439; doi:10.3897/phytokeys.261.157043)
Supplement: Supplementary material 1 — Specimens examined of Ecuadorian Nototriche apart from N.antisanensis, the latter listed in the text [file phytokeys-261-001_article-157043__-s001.docx]

Supplementary Material 1. Specimens examined of Ecuadorian *Nototriche* apart from *N. antisanensis*, the latter listed in the text.

**1. *Nototriche ecuadoriensis* Fryxell.**

**Ecuador. Napo:** Páramo de Papallacta, sector El Paso, 00°22'S, 78°08'W, 4060 m elev., 28 Oct 1984 (fl), *A. Freire-Fierro 33* (QCA); Antisana, sitio de experimentación Antisana, en el camino que va desde la laguna de Santa Lucía hacia el Glaciar 17, 00°28'0.696 ''S, 78°9'45.503''W, 4300-4600 m elev., 5 Aug 2013 (fl), *J. Irazábal & Varela F. 378* (QCA); Laguna de Hoyas, páramo de Papallacta, 0°15'S, 78°12'W, 4200 m elev., 8 Aug 1987, *P.M. Ramsay & P.J. Merrow-Smith 192* (QCA, QCNE); Antisana, alrededores de la Laguna de Santa Lucía, sitio de experimentos GLORIA cercano al glaciar, 4567 m elev., 8 Dec 2024, *E. Trujillo et al. 38* (QCA), Ibid., *E. Trujillo et al. 39* (QCA); Colecciones dentro del Área de Conservación Hídrica Antisana, 0°28'31.8''S, 78°13'13.2''W, 4256 m elev., 22 Oct 2019 (fl), *R. Zambrano-Cevallos & A. Villareal 517-B* (QCA).

**2. *Nototriche jamesonii* A.W.Hill**

**Ecuador. Bolívar:** 28–29 km NE of Guaranda on the Guaranda-Ambato hwy, 01°36’S, 79°00’W, 4185 m elev., 25 Jun 1989 (fl), *L.J. Dorr & I. Valdespino 6482* (QCA, QCNE); Puna El Arenal, ca. de la base del Chimborazo, km 60 vía Ambato-Guaranda, 01°25’56,9’’S, 78°54’30.3’’W, 4177 m elev., 6 Apr 2017, *I. Fragoso-Martínez et al. 616* (QCNE); Superpáramo area W of Volcán Chimborazo, ca. 33 km N of Guaranda, 01°30’S, 78°56’W, 4120 m elev., 29 Nov 1989 (fl), *J.L. Luteyn 13397* (QCA, QCNE); Area W of Volcán Chimborazo, ca. 33 km N of Guaranda, 01°30'S, 78°56'W, 4120 m elev., 16 Jan 1985 (fl), *J.L. Luteyn & E. Cotton 11080* (QCA). **Chimborazo:** Reserva Faunística Chimborazo, Páramo, Bosque *Polylepis* y alrededores, 01°32’S, 78°53’W, 4100 m elev., 9 Sep 2006 (fl), *J. Caranqui et al. 1586* (QCNE); Páramo del Chimborazo, 01°28'S, 78°52'W, 4260 m elev., 24 Jul 2006 (fl), *A. Castillo 10* (QCA); Reserva Faunística del Chimborazo, parroquia San Juan, puna, arenal del Chimborazo, lado occidental, 01°28’S, 78°52’W, 4260 m elev., 4 Jul 1992 (fl), *C. Cerón 19313* (QCNE); Base of road to, and 7 km from the refugio Chimborazo, 01°27’S, 78°50’W, 4620 m elev., 23 Jun 1989 (fl), *L.J. Dorr & I. Valdespino 6437* (QCA, QCNE); Arenales del Chimborazo, 01°28'S, 78°48'W, 4300 m elev., 19 Oct 2000 (fl), *L. Endara et al. 361* (QCA); Guaranda-Ambato road, below Mt. Chimborazo, 01°35'18''S, 78°59'58''W, 4160 m elev., 6 Jul 1984 (fl), *J. Grimes & C. Todzia 2569* (QCA); Arenales del Chimborazo, 01°30'S, 78°51'W, 4000 m elev., 31 May 2002 (fl), *S. León-Yánez & K. Romoleroux 1967* (QCA); Upper WSW slopes of Volcán Chimborazo, 01°28'S, 78°50'W, 4750 m elev., 7 Feb 1988 (fl), *U. Molau & B. Eriksen 2986* (QCA); South-Western slope of volcano Chimborazo, 01°01'S, 78°46'W, 3800-3900 m elev., 26 Jun 2012 (fl), *N. Morueta-Holme et al. 26* (QCA); Cantón Guano, ladera W del Volcán Chimborazo, 4425 m elev., 30 Dec 2011, *G. Peyre & J. Castillo 58* (QCA); W side of the Chimborazo volcano, 01°28'S, 78°52'W, 4600–4800 m elev., 5 Jul 1999 (fl), *P. Sklenar 7537* (QCA); Volcan Chimborazo, W side of the mountain, 1°28’S, 78°48’W, 4700 m elev., 14 Sep 1995 (fl), *P. Sklenar & V. Kosteckova 140-1* (QCNE).

**3. *Nototriche phyllanthos* (Cav.) A.W.Hill**

**Ecuador. Cotopaxi:** Paramo around the Illiniza peaks, 4 mi W of town of Magdalena, 00°39’S, 78°40’W, 4600 m elev., 2 Apr 1991 (fl), *R. Bensman 362* (QCNE); Páramo de Quispicacha, summit plateau of Loma Pucyucuchu, 01°05’00’’S, 78°50’30’’W, 4370–4420 m elev., 23 Oct 2006, *P. Sklenar 9133* (QCA), Ibid., 01°05’09’’S, 78°50’42’’W, 4500–4545 m elev., 25 Oct 2006 (fl), *P. Sklenar 9150* (QCA); Páramo de Zumbagua, 00°08’S, 78°54’W, 4000 m elev., 21 Jun 1986 (fl), *N. Urgilés 19* (QCA). **Napo:** Falda occ. del Cerro Antisana, origen del río Antisana, 00°28’S, 78°12’W, 4200 m elev., 27–28 Jan 1983, *H. Balslev et al. 3946* (QCA); Near the Summit of Cerro Puntas, 00°12’S, 78°12’W, 4450 m elev., 9 Jun 1985 (fl), *J. Bosco & M.C. Marcillo 214* (QCA); Páramo de Guamaní, small peak c. 6 km south of Paso de la Virgen, 00°21’S, 78°13’W, 4200–4250 m elev., 28 Nov 1985 (fl), *S. Laegaard 55720* (QCA); SW slopes of Volcán Antisana, 2–3 km N of Hacienda El Hato and 5–6 km NNE of Laguna Micacocha, 00°28’S, 78°09’W, 4,200–4,300 m elev., 1 Jul 1979 (fl), *B. Lojtant & U. Molau 15388* (QCA); Reserva Ecológica Antisana, Southwest slopes of Volcán Antisana, 100 m below the glacier, 00°29’28’’S, 78°09’34’’W, 4,700 m elev., 28 Nov 1998 (fl), *D.A. Neill et al. 11494* (QCNE); Volcán Antisana, rocky slopes on the W side of the mountain, 00°30’S, 78°10’W, 4500–4550 m elev., 21 Jul 1997 (fl), *P. Sklenar & V. Sklenarova 2792* (QCA); Cantón Quijos, Área de Conservación Hídrica Antisana, 0°28’31.8’’S, 78°13’13.2’’W, 4256 m elev., 22 Oct 2019 (fl), *R. Zambrano–Cevallos & A. Villareal 517–A* (QCA). **Pichincha:** Faldas sur–este, volcán Guagua Pichincha, 0°10’S, 78°35’W, 4500 m elev., 25 May 1985 (fl), *J. Bosco & M. Marcillo 47* (QCA); Páramo, ca. 3 km NE of the volcano Iliniza Sur, 00°24’S, 78°42’W, 4000–4600 m elev., 19 Mar 1995 (fl), *J.L. Clark 470* (QCNE); Cantón Quito, Volcán Guagua Pichincha, 00°10’S, 78°35’W, 3500 m elev., 21 Apr 1996 (fl), *J.L. Clark 2522* (QCNE); Quito, Rucu Pichincha, along Trail between Summit and Loma de las Antenas, 00°78’S, 78°30’W, 2800 m elev., 10 Sep 1995 (fl), *J.L. Clark & S. Fishman 1456* (QCNE); Volcán Guagua Pichincha, Cima Padre Encantado, 00°08’24.58’’S, 78°47’28.2’’W, 4500 m elev., 16 Nov 2014, *S.A. Duchicela et al. 22* (QCA); Cantón Quito, Parroquia Lloa, Sector Noreste del Volcán Guagua Pichincha, 0°10’30’’S, 78°35’45’’W, 4563 m elev., 12 Aug 2014 (fl), *D. Fernández et al. 4563* (QCNE); Carretero Cayambe–Monjas–El Refugio, 0°2’N, 78°2’W, 4540 m elev., 3 Dec 1993, *A. Freire–Fierro et al. 2587* (QCA, QCNE); Filo sur de Co. Sincholagua, 00°32’S, 78°23’W, 4,340 m elev., 23 Dec 1979 (fl), *H. QCA 1980* (QCA); Reserva Yanacocha, flada y cumbre del cerro Ingapirca, 00°08’S, 78°34’W, 4200 m elev., 18 Oct 2014 (fl), *R.J. Irazábal & D. Ponce 272* (QCA); Páramo de Guamaní, Carretera Pifo–Papallacta, km 27, 00°10’42.34’’S, 78°35’48.60’’W, 4,415 m elev., 13 Jan 1990 (fl), *S. León–Yánez 1480* (QCA); Upper SE slopes of Guagua Pichincha, between the refuge and the cráter rim, 00°11’S, 78°36’W, 4450–4650 m elev., 9 Jan 1988 (fl), *U. Molau et al. 2387* (QCA QCNE); Guagua Pichincha, around the Refugio above Lloa, 00°11’S, 78°33’W, 4400–4500 m elev., 18 Nov 1990 (fl), *B. Ollgaard 98261* (QCA, QCNE); Volcan Rucu Pichincha, NE side of the mountain, along the trail from Teleférico to the summit, 00°09’S, 78°34’W, 4500 m elev., 6 Jul 2007 (fl), *P. Sklenar 10001* (QCA); NE slopes of Rucu Pichincha, 00°10’S, 78°34’W, 4500 m elev., 13 May 1995 (fl), *P. Sklenar & V. Kosteckova 7–3* (QCNE), Ibid., 0°10’S, 78°34’W, 4600 m elev., 18 May 1995 (fl), *P. Sklenar & V. Kosteckova 12–13* (QCNE); Cantón Cayambe, Nevado Cayambe, SW side, 0°01’N, 78°01’W, 4400–4500 m elev., 2 Jul 1995 (fl), *P. Sklenar & V. Kosteckova 745* (QCNE); Cayambe volcano, near the refuge, 00°00’23’’N, 78°01’07’’W, 4395 m elev., 6 Oct 2012 (fl), *C. Ulloa et al. 2412* (QCA).

***4. Nototriche hartwegii* A.W.Hill**

**Ecuador. Azuay:** Parque Nacional Cajas, road Cuenca-Sayahuasí-Molleturo km 36, trail to Paraguillas, at the pass, 02°46’19’’S, 79°14’37’’W, 4100–4300 m elev., s.d., *P.M. Jørgensen et al.* *2422* (QCA, QCNE); Páramo del Cajas, en el paso, 02°46'S, 79°14'W, 4000–4200 m elev., 27 Aug 1985 (fl), *S. Laegaard* *55075* (QCA); Cajas National Park, E flanks and summit area of Cerro Amarillo, 02°45'S, 79°15'W, 4300–4400 m elev., 13 Jul 1997, *P. Sklenar & V. Sklenarova* *2488* (QCA); Parque Nacional Cajas, road Cuenca-Sayahuasí-Molleturo km 36, trail to Paraguillas, at the pass, 02°46'10.6''S, 79°14'35.9''W, 4300 m elev., 21 Nov 2010, *P. Sklenar, V. Zeisek* *13054* (QCA); Parque Nacional Cajas, Vía Cuenca-Molleturo, mirador "Tres Cruces", sendero Paraguillas, 02°46’14’’S 79°14’37’’W, 4310 m elev., 29 Oct 2024, *E.J. Trujillo et al.* *14* (QCA), Ibid., *E.J. Trujillo et al.* *16* (QCA); Parque Nacional Cajas, km 35.7 redondel Cuenca-Molleturo, en el paso, sendero Paraguillas, 02°46’39’’S, 79°14’30’’W, 4100–4180 m elev., s.d., *C. Ulloa et al.* *1184* (QCNE). **Chimborazo:** Laguna de Ozogoche, 02°13'S, 78°37'W, 3500 m elev., 10 Jul 2013, *J. Caranqui* *2453* (QCA).
